# Changes in Outcomes of Hepatic Cytoreduction for Neuroendocrine Liver Metastases over Two Decades: From Major Hepatectomy to Parenchyma-Sparing Approaches

**DOI:** 10.1245/s10434-026-20383-z

**Published:** 2026-08-12

**Authors:** Kyoji Ito, Mikel Madi, Jonathan J. Hue, Elsa Melissa Arvide, Adriana Gamboa, Timothy E. Newhook, Yun Shin Chun, Hop S. Tran Cao, Ching-Wei D. Tzeng, Arvind Dasari, James C. Yao, Chandrikha Chandrasekharan, Matthew H. G. Katz, Jean-Nicolas Vauthey, Jessica E. Maxwell

**Affiliations:** 1 Department of Surgical Oncology, The University of Texas MD Anderson Cancer Center, Houston, TX USA; 2 Department of Gastrointestinal Medical Oncology, The University of Texas MD Anderson Cancer Center, Houston, TX USA

**Keywords:** Neuroendocrine neoplasm, Liver metastasis

## Abstract

**Background:**

Surgical and systemic management of neuroendocrine liver metastases (NELM) has evolved over the past decades. This study aimed to evaluate temporal changes in prognosis and identify factors associated with improved survival after hepatic cytoreduction for NELM.

**Patients and Methods:**

Patients who underwent hepatic cytoreduction for NELM during 1998–2023 were retrospectively analyzed and divided into earlier period (EP, 1998–2008) and later period (LP, 2009–2023) cohorts. Clinicopathological features, perioperative outcomes, and survival were compared. Covariate-adjusted survival curves and propensity score matching were used to compare overall survival (OS) between major hepatectomy and liver-parenchyma-sparing cytoreduction (LPSC).

**Results:**

The study included 293 patients, 132 in the EP cohort and 161 in the LP cohort. The LP cohort had a lower rate of major hepatectomy (32.9% versus 60.0%, *P* < 0.001) and significantly better OS (10-year OS rate, 67.4% versus 44.2%, *P* < 0.001). Major hepatectomy remained independently associated with worse OS after adjustment for treatment period and other covariates [hazard ratio (HR) 1.74, 95% confidence interval (CI) 1.02–2.98, *P* = 0.043]. This association was consistent after propensity score matching. LP patients received local therapy (including surgery, percutaneous ablation, and transarterial embolization), somatostatin analogs, and targeted agents more frequently than EP patients after tumor progression.

**Conclusions:**

OS after hepatic cytoreduction for NELM improved during 1998–2023 in parallel with a shift from extensive resections to a parenchyma-sparing approach and the integration of modern systemic therapies. These findings support individualized cytoreductive strategies that balance tumor reduction with preservation of liver parenchyma and future treatment options.

**Supplementary Information:**

The online version contains supplementary material available at https://doi.org/10.1245/s10434-026-20383-z.

Neuroendocrine neoplasms (NENs) are relatively rare tumors that frequently give rise to liver metastases [neuroendocrine liver metastases (NELM)], which have a significant negative impact on prognosis.^1–3^ Surgical resection has played an important role in achieving long-term survival in patients with NELM.^4,5^ Traditionally, maximizing cytoreduction was considered the primary goal of surgical resection, and achieving a residual tumor volume of less than 10% (i.e., > 90% cytoreduction) was regarded as the benchmark for improved outcomes.^6,7^ Consequently, many patients with NELM underwent major hepatectomy;^8,9^ however, the limited preservation of liver parenchyma often restricted the feasibility of repeat hepatectomy or local therapy in the event of tumor progression.^10^

Recent studies have demonstrated that the residual tumor volume is the key prognostic determinant in patients undergoing hepatic cytoreduction for NELM and that achieving ≥ 70% cytoreduction may provide survival benefits similar to those observed with 90% cytoreduction.^11,12^ Because of these findings, liver-parenchyma-sparing cytoreduction (LPSC) is increasingly being used instead of major hepatectomy as LPSC preserves hepatic reserve during the initial operation and maintains the potential for repeat resection or local therapy upon tumor progression.^11,13^

Over the past 25 years, systemic therapies for NENs have also evolved significantly. Somatostatin analogs (SSAs), molecular targeted agents, and peptide receptor radionuclide therapy (PRRT) have been sequentially introduced^14–18^ and have contributed to disease control and maintenance of performance status after liver resection. These advances in systemic therapy, together with the evolution of surgical strategies, are thought to have contributed to the improvement of long-term outcomes in patients with NELM.

This study aimed to evaluate temporal changes in prognosis and identify factors associated with improved survival among patients who underwent hepatic cytoreduction for NELM during 1998–2023. We specifically evaluated temporal changes in surgical strategy, including the relative use of major hepatectomy and LPSC, and examined the association between surgical strategy and overall survival (OS).

## Patients and Methods

### Patient Selection and Data Collection

The Institutional Review Board of The University of Texas MD Anderson Cancer Center approved this retrospective study (#2023-0433). From a prospectively maintained database, we included all patients who underwent liver surgery including debulking cytoreduction for NELM between January 1998 and December 2023, with follow-up through March 2025. We divided the patients into those treated during an earlier period (EP, 1998–2008) and those treated during a later period (LP, 2009–2023). The cutoff was set at 2009 because this was the year of publication of the landmark PROMID trial^14^ showing evidence for the antitumor effect of somatostatin analogs, the first of several systemic treatment options to be shown effective for treatment of NELM.^14–18^

### Institutional Approach to Management of NELM

During the study period, the MD Anderson approach to management of NELM was as follows. Preoperative imaging included chest computed tomography and abdominopelvic imaging, including computed tomography or magnetic resonance imaging, in all patients. In addition, starting in 2004, preoperative radionucleotide scintigraphy (i.e., octreotide scan or gallium Ga 68 dotatate positron emission tomography) was considered, particularly in patients with unknown primary tumors or suspected extrahepatic metastases. NELM were deemed resectable when preservation of ≥ 30% of the total liver volume standardized to the patient’s body surface area, with sparing of two contiguous hepatic segments and maintenance of vascular inflow, vascular outflow, and biliary drainage, could be achieved.^19^ For patients with an anticipated insufficient future liver remnant, prehepatectomy portal vein embolization and/or staged hepatectomy was considered. Surgical cytoreduction was performed to clear the liver of disease if possible, but incomplete cytoreduction was allowed if at least 70–90% of the tumor burden could be resected. Indications for surgical cytoreduction were determined not by anatomic resectability alone, but by multidisciplinary assessment of performance status, operative risk, tumor biology, presence and controllability of extrahepatic disease, and feasibility of achieving meaningful cytoreduction. Ablation was performed as an adjunct to resection for lesions deep within the parenchyma. Ablation was not performed for metastases adjacent to major biliary structures, including metastases at the hepatic duct confluence. Patients were routinely followed after cytoreduction with history, physical examination, laboratory evaluation, and imaging every 3–6 months.

### Pharmacologic Therapies

SSAs such as octreotide or lanreotide were administered to patients with functional tumors, primarily for the control of carcinoid syndrome. SSAs were also considered for patients with nonfunctional, aggressive tumors to control proliferation, both in the preoperative and postoperative settings. For patients with well-differentiated NENs [grade 1 or 2 neuroendocrine tumor (NET)] with extrahepatic metastases or for whom 100% disease clearance was not feasible, perioperative pharmacologic therapies were often used. SSAs were commonly used as first-line therapy. For patients with more aggressive disease, systemic therapies such as 5-fluorouracil, doxorubicin, streptozocin, capecitabine, or temozolomide or molecular therapies such as everolimus or sunitinib could be offered. No patients in this cohort were treated with PRRT prior to surgery. The absence of preoperative PRRT reflected institutional practice during the study period, in which surgical cytoreduction was generally considered before PRRT for patients deemed suitable for surgery. For patients with poorly differentiated neuroendocrine neoplasm (neuroendocrine carcinoma), perioperative chemotherapy with cisplatin plus etoposide was administered.

### Definition

The extent of hepatic cytoreduction was abstracted from the operative report and reflected the operating surgeon’s assessment of the proportion of tumor burden removed at the completion of surgery. Postoperative morbidity and 90-day mortality were recorded. Major morbidity was defined as Clavien–Dindo grade III or higher, and 90-day mortality was defined as death from any cause within 90 days after liver surgery.

### Statistical Analysis

Clinicopathological data were compared between groups. Continuous variables were presented as median and interquartile range (IQR) and compared using the Mann–Whitney *U* test. Categorical variables were presented as number (percentage) and compared using the *χ*^*2*^ test. OS and progression-free survival (PFS) curves were constructed using the Kaplan–Meier method and compared using the log-rank test. Deaths without progression were censored for the PFS analysis. Univariable and multivariable analyses for OS and PFS were conducted by Cox regression model. Hazard ratios (HRs) with the respective 95% confidence intervals (CIs) were calculated for each factor on univariable analyses. Factors with *P* < 0.1 on univariable analysis were advanced to the multivariable regression model. Covariate-adjusted survival curves comparing OS between patients who underwent major hepatectomy and those who underwent LPSC were generated from this multivariable model.

To identify the association between operative procedure for NELM (major hepatectomy versus LPSC) and OS, propensity score matching was performed to create patient groups with similar baseline characteristics. To balance the background factors, the propensity score model was developed using a logistic regression model adjusted for age, body mass index, primary tumor site, number and size of liver metastases, and World Health Organization grade. A 1:1 match without replacement was performed using logit (propensity score) through the nearest available match with the caliper set at 0.15. Covariate balance before and after matching was assessed using absolute standardized mean differences. Balance was considered acceptable when all absolute standardized mean differences were < 0.10.

Statistical analyses were performed using R version 4.3.1 (R Foundation for Statistical Computing, Vienna, Austria).

## Results

### Patient Characteristics

The study included 293 patients, 132 (45.1%) in the EP cohort and 161 (54.9%) in the LP cohort. The patients’ clinicopathologic characteristics are summarized in Table 1. The median age was 56.9 years, and 150 patients (51.2%) were male. The most common primary tumor sites were small intestine (*n* = 134, 45.7%) and pancreas (*n* = 100, 34.1%). Among patients with available World Health Organization grade data, 88.1% had grade 1 or 2 NET, 8.5% had grade 3 NET, and 3.5% had poorly differentiated NET (neuroendocrine carcinoma). The mean number of liver lesions was similar between the EP and LP cohorts (4.0 ± 4.8 versus 4.0 ± 4.7; *P* = 0.974), as was the mean maximum diameter of liver metastasis (39.9 ± 27.0 mm versus 36.8 ± 34.3 mm; *P* = 0.407). Compared with the EP cohort, the LP cohort had a lower median (IQR) maximum diameter of liver metastasis [25.0 mm (15.0–44.0 mm) versus 34.0 mm (20.0–55.0 mm), *P* = 0.024], lower rate of major hepatectomy (32.9% versus 60.0%, *P* < 0.001), and lower rate of concomitant ablation (11.8% versus 27.3%, *P* = 0.001). The 90-day mortality was low and did not differ between the LP and EP cohorts (1.2% versus 1.5%; *P* > 0.999).

**Table 1:** Demographic and clinicopathologic characteristics^a^

Characteristic	All patients (*n* = 293)	EP, 1998–2008 (*n* = 132)	LP, 2009–2023 (*n* = 161)	*P*-value
*Patient factors*				
Age, median (IQR), years	56.9 (49.4–65.3)	56.8 (48.4–64.4)	57.0 (49.8–65.8)	0.726
Sex, male/female	150/143	64/68	86/75	0.47
BMI, median (IQR), kg/m^2^	27.3 (24.3–31.2)	26.7 (23.7–30.5)	27.7 (24.6–31.9)	0.135
*Primary tumor factors*				
Primary lesion site				0.746
Small intestine	134 (45.7)	65 (49.2)	69 (42.9)	
Pancreas	100 (34.1)	39 (29.5)	61 (37.9)	
Colon/rectum	14 (4.8)	7 (5.3)	7 (4.3)	
Lung	13 (4.4)	7 (5.3)	6 (3.7)	
Other^b^	14 (4.8)	6 (4.5)	8 (5.0)	
Unknown	18 (6.1)	8 (6.1)	10 (6.2)	
Lymph node metastases	180 (81.1)^d^	79 (85.9)^e^	101 (77.7)^f^	0.174
*Liver tumor factors*				
Synchronous metastases	175 (64.3)^d^	71 (57.7)^e^	104 (69.8)^f^	0.115
Extrahepatic metastases	12 (4.1)	7 (5.3)	5 (3.1)	0.733
Peritoneum	5 (1.7)	3 (2.3)	2 (1.2)	
Pancreas	2 (0.7)	1 (0.8)	1 (0.6)	
Other^c^	5 (1.7)	3 (2.3)	2 (1.2)	
Tumor number, median (IQR)	2.0 (1.0–5.0)	2.0 (1.0–5.0)	2.0 (1.0–5.0)	0.607
Maximum tumor diameter, median (IQR), mm	30.0 (17.0–50.0)	34.0 (20.0–55.0)	25.0 (15.0–44.0)	0.024
Prehepatectomy chemotherapy	101 (34.5)	41 (31.1)	60 (37.3)	0.323
Cytotoxic chemotherapy	47 (16.1)	23 (17.4)	24 (15.0)	0.688
Molecular targeted therapy	12 (4.1)	5 (3.8)	7 (4.3)	1
Somatostatin analogue	58 (19.8)	19 (14.4)	39 (24.2)	0.051
Posthepatectomy chemotherapy	34 (11.6)	13 (9.8)	21 (13.0)	0.505
*Resection factors*				
Major hepatectomy	129 (45.3)^d^	78 (60.0)^e^	51 (32.9)^f^	< 0.001
Concomitant ablation	55 (18.8)	36 (27.3)	19 (11.8)	0.001
Extent of resection				> 0.999
100%	267 (91.1)	120 (90.9)	147 (91.3)	
≥ 90%	20 (6.8)	7 (5.3)	13 (8.1)	
≥ 70, < 90%	2 (0.7)	2 (1.5)	0 (0.0)	
< 70%	4 (1.4)	3 (2.3)	1 (0.6)	
WHO grade				0.438
Grade 1/2 NET	177 (88.1)^d^	47 (88.7)^e^	130 (87.8)^f^	
Grade 3 NET	17 (8.5)^d^	3 (5.7)^e^	14 (9.5)^f^	
NEC	7 (3.5)^d^	3 (5.7)^e^	4 (2.7)^f^	

^a^Values in table are number of patients (percentage) unless otherwise indicated

^b^Other includes one case each of bone, lymph node, stomach, testis, and eyeball origin

^c^Other includes one case each of bone, stomach, and testis origin

^d^Data not available for lymph node metastases in 71 patients, synchronous metastases in 21 patients, major hepatectomy in 8 patients, and WHO grade in 92 patients

^e^Data not available for lymph node metastases in 40 patients, synchronous metastases in 9 patients, major hepatectomy in 2 patients, and WHO grade in 79 patients

^f^Data not available for lymph node metastases in 31 patients, synchronous metastases in 12 patients, major hepatectomy in 6 patients, and WHO grade in 13 patients

*BMI* body mass index, *EP* earlier period, *IQR* interquartile range, *LP* later period, *NEC* neuroendocrine carcinoma, *NET* neuroendocrine tumor, *WHO* World Health Organization

### Oncologic Outcomes

**Fig. 1:**
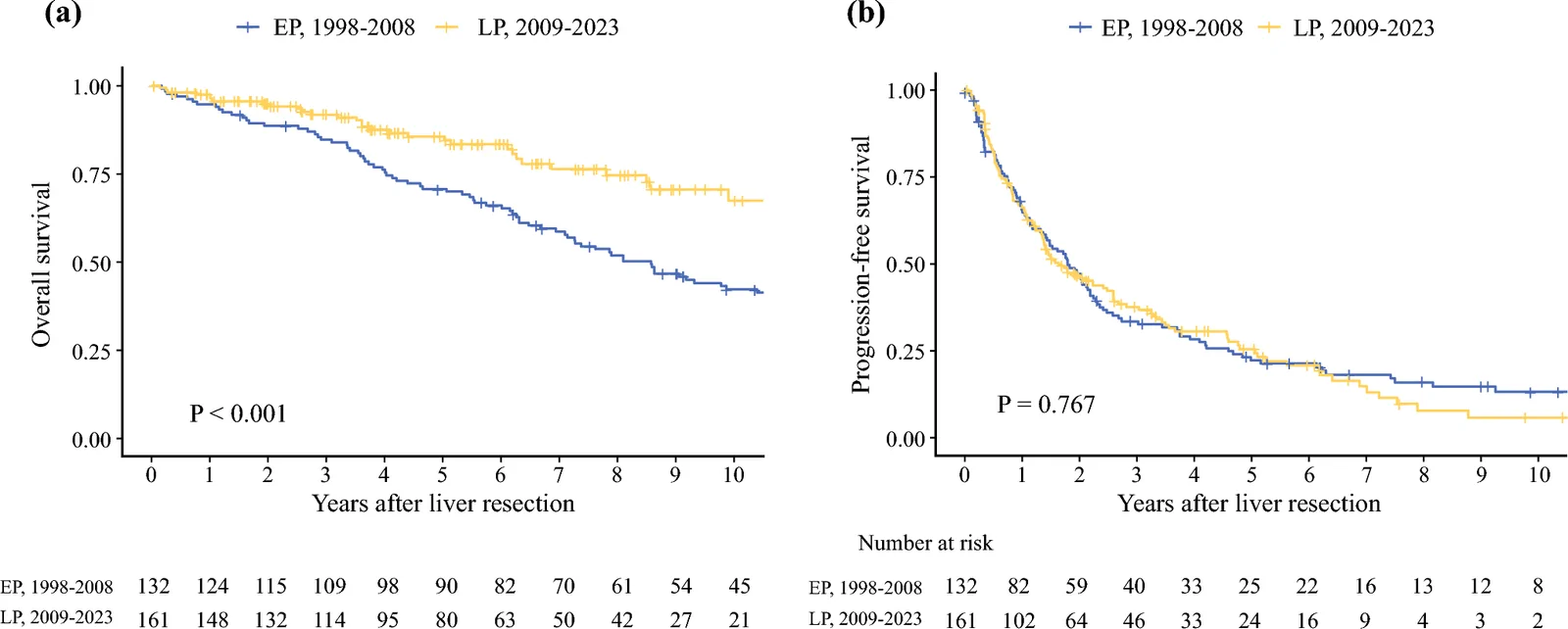
Overall survival (**a**) and progression-free survival (**b**) stratified by the earlier period (EP) and the later period (LP)

After a median (IQR) follow-up period of 6.0 years (3.2–6.9), the median OS was 10.7 years (95% CI 9.1–12.2). A total of 127 patients died during the observation period. OS was significantly better in the LP cohort than in the EP cohort (*P* < 0.001; Fig. 1A). The median survival was not reached in the LP cohort and was 8.6 years (95% CI 7.1–10.7) in the EP cohort, and the 10-year survival rate was 67.4% in the LP cohort and 44.2% in the EP cohort. There was no difference in PFS between the two cohorts (*P* = 0.767, Fig. 1B).

Multivariable analysis of OS showed that older age (HR 1.04, 95% CI 1.02–1.06, *P* = 0.001), maximum diameter of liver metastasis > 5 cm (HR 2.21, 95% CI 1.23–3.97, *P* = 0.008), major hepatectomy (HR 1.74, 95% CI 1.02–2.98, *P* = 0.043), grade 3 NET (HR 5.81, 95% CI 2.35–14.33, *P* < 0.001), and NEC (HR 4.62, 95% CI 1.48–14.43, *P* = 0.008) were independently associated with worse OS, whereas treatment during the LP was independently associated with better OS (HR 0.52, 95% CI 0.29–0.93, *P* = 0.028) (Table 2). Multivariable analysis of PFS showed that tumor number ≥ 2 (HR 1.6, 95% CI 1.08–2.37, *P* = 0.019), maximum diameter of liver metastasis > 5 cm (HR 1.72, 95% CI 1.16–2.55, *P* = 0.007), and incomplete (≥ 90%) hepatic cytoreduction (HR 2.09, 95% CI 1.19–3.66, *P* = 0.01) were worse prognostic factors, while primary small intestinal disease (HR 0.55, 95% CI 0.37–0.81, *P* = 0.003) was a better prognostic factor (Supplementary Table 1).

**Table 2:** Multivariable analysis of overall survival^a^

Variable	Univariable analysis			Multivariable analysis		
	HR	95% CI	*P*-value	HR	95% CI	*P*-value
*Patient factors*						
Age, years	1.02	1.01–1.04	0.006	1.04	1.02–1.06	0.001
Sex, male:female	0.97	0.68–1.38	0.876			
BMI, kg/m^2^	0.96	0.93–0.99	0.017	0.95	0.90–1.00	0.06
Primary tumor factors						
*Primary lesion site*						
Pancreas	1 (Ref)	–	–	1 (Ref)	–	–
Small intestine	0.51	0.35–0.74	< 0.001	0.58	0.30–1.12	0.107
Other	1.62	1.06–2.48	0.025	0.67	0.34–1.34	0.259
Lymph node metastasis	1.2	0.66–2.16	0.555			
*Liver tumor factors*						
Synchronous metastases	0.9	0.62–1.31	0.589			
Extrahepatic metastases	1.35	0.58–3.15	0.48			
Tumor number ≥ 2	1.04	0.69–1.55	0.863			
Maximum tumor diameter > 5 cm	1.66	1.14–2.41	0.008	2.21	1.23–3.97	0.008
Prehepatectomy chemotherapy	1.22	0.84–1.78	0.29			
*Resection factors*						
Major hepatectomy	1.37	0.96–1.95	0.08	1.74	1.02–2.98	0.043
Concomitant ablation	1.1	0.73–1.67	0.643			
*Extent of resection*						
100%	1 (Ref)	–	–			
≥ 90%	0.96	0.45–2.05	0.909			
≥ 70, < 90%	1.63	0.40–6.62	0.493			
< 70%	0.53	0.12–2.22	0.383			
*WHO grade*						
Grade 1/2 NET	1 (Ref)	–	–	1 (Ref)	–	–
Grade 3 NET	4.1	1.97–8.52	< 0.001	5.81	2.35–14.33	< 0.001
NEC	2.92	1.05–8.11	0.04	4.62	1.48–14.43	0.008
*Treatment period*						
EP (1998–2008)	1 (Ref)	–	–	1 (Ref)	–	–
LP (2009–2023)	0.49	0.32–0.73	< 0.001	0.52	0.29–0.93	0.028

^a^196 patients were included in the multivariate analysis of overall survival

*BMI* body mass index, *EP* earlier period, *LP* later period, *NEC* neuroendocrine carcinoma, *NET* neuroendocrine tumor, *WHO* World Health Organization

### Covariate-Adjusted Survival Analysis

**Fig. 2:**
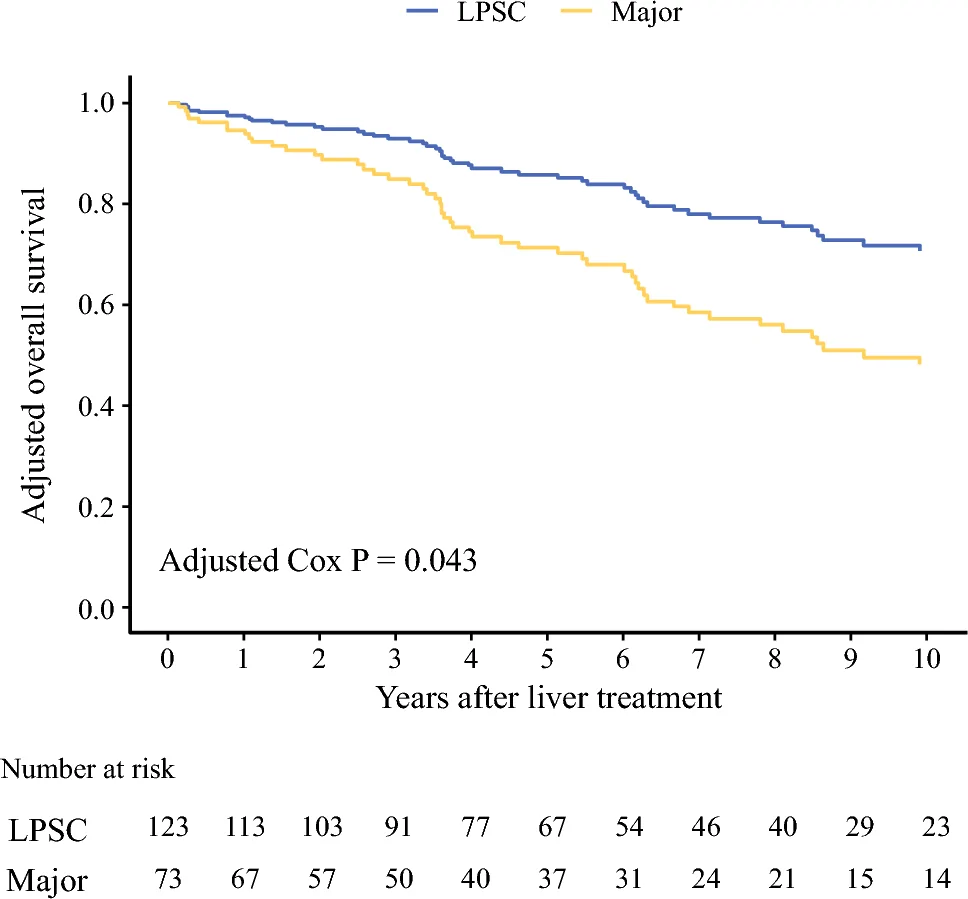
Covariate-adjusted survival curves comparing overall survival between patients who underwent major hepatectomy and those who underwent liver-parenchyma-sparing cytoreduction; curves were generated from the multivariable Cox model presented in Table 2

Covariate-adjusted survival curves were generated from the multivariable Cox model presented in Table 2 to compare OS between patients who underwent major hepatectomy and those who underwent LPSC. The covariate-adjusted survival curves showed worse OS among patients who underwent major hepatectomy than among those who underwent LPSC; the adjusted 10-year OS estimates were 54.1% and 67.7%, respectively (adjusted HR 1.74; 95% CI 1.02–2.98; *P* = 0.043; Fig. 2).

### Comparison between Major Hepatectomy and LPSC

Before matching, patients who underwent major hepatectomy had several differences in baseline characteristics compared with those who underwent LPSC. The major hepatectomy group was slightly younger (mean age, 55.1 versus 57.9 years; *P* = 0.043), had a different distribution of primary tumor sites (*P* = 0.035), and had greater hepatic tumor burden, including a higher tumor number (mean, 5.0 versus 3.3; *P* = 0.003) and larger maximum tumor diameter (mean, 49.0 versus 29.7 mm; *P* < 0.001).

After 1:1 matching, 50 patients in the major hepatectomy group and 50 patients in the LPSC group were analyzed. Covariate balance improved substantially between groups after matching; all absolute standardized mean differences were below 0.10 except for small intestine primary tumor site, for which a small residual imbalance remained (absolute standardized mean difference 0.129) (Supplementary Table 3, Supplementary Fig. 1). In the matched analysis of OS, patients who underwent major hepatectomy had significantly worse survival than those who underwent LPSC (10-year OS rate, 54.8% versus 73.9%; *P* = 0.019) (Supplementary Fig. 2).

Postoperative outcomes did not significantly differ between the two groups either before or after matching. Before matching, overall morbidity, major morbidity, and 90-day mortality were comparable between the major hepatectomy and LPSC groups (47.3% versus 39.1%, *P* = 0.267; 19.4% versus 10.3%, *P* = 0.064; and 1.6% versus 1.3%, *P* > 0.999, respectively). After matching, these outcomes remained similar between the major hepatectomy and LPSC groups (overall morbidity, 44.0% versus 44.0%, *P* > 0.999; major morbidity, 10.0% versus 10.0%, *P* > 0.999; and 90-day mortality, 2.0% versus 0%, *P* > 0.999).

### Treatment for Tumor Progression after NELM Cytoreduction

Initial treatments administered to patients with tumor progression after NELM cytoreduction are presented in Table 3. Compared with the EP cohort, the LP cohort had higher rates of local therapy (including surgery, percutaneous ablation, and transarterial embolization) (32.2% versus 25.3%), SSA (44.4% versus 38.6%), and molecular targeted therapy (15.6% versus 9.6%), and a lower rate of cytotoxic chemotherapy (5.6% versus 16.9%). In the multivariable analysis of OS in patients who received treatment for tumor progression, older age (HR 1.05, 95% CI 1.01–1.08, *P* = 0.012) and grade 3 NET (HR 5.46, 95% CI 1.68–17.72, *P* = 0.005) were independently associated with worse OS, and local therapy for initial treatment (HR 0.41, 95% CI 0.17–0.97, *P* = 0.043) was independently associated with better OS (Supplementary Table 2).

**Table 3:** Treatment methods for patients treated for tumor progression

Treatment	All patients (*n* = 173)	EP, 1998–2008 (*n* = 83)	LP, 2009–2023 (*n* = 90)	*P*-value
				0.022
Local therapy^a^	50 (28.9)	21 (25.3)	29 (32.2)	
Somatostatin analogue	72 (41.6)	32 (38.6)	40 (44.4)	
Molecular targeted therapy	22 (12.7)	8 (9.6)	14 (15.6)	
Cytotoxic chemotherapy	19 (11.0)	14 (16.9)	5 (5.6)	
Other	10 (5.8)	8 (9.6)	2 (2.2)	

^a^Surgical resection, percutaneous ablation, and transarterial embolization

*EP* earlier period, *LP* later period

## Discussion

This study demonstrates that OS after hepatic cytoreduction for NELM improved during 1998–2023, a period characterized by both a shift toward parenchyma-sparing approaches and advances in systemic therapy. Hepatic cytoreduction during the LP, which was most often performed using a parenchyma-sparing approach, was significantly associated with better OS than hepatic cytoreduction during the EP, when the majority of patients underwent major hepatectomy. Multivariable analysis including treatment period identified major hepatectomy as an independent predictor of poor prognosis. In addition, covariate-adjusted survival curves showed worse OS among patients who underwent major hepatectomy than among those who underwent LPSC. This association remained consistent even after balancing baseline characteristics between the major hepatectomy and LPSC groups using propensity score matching. These findings suggest that the decreased use of major hepatectomy may have contributed, at least in part, to improved OS, although this association should be interpreted in the context of concurrent advances in systemic therapy and patient selection over time.

PFS did not differ significantly between the EP and LP cohorts. Among patients who received treatment for disease progression after resection, the use of local therapy as the initial treatment was an independent favorable prognostic factor. This association may reflect favorable tumor biology with low-volume disease in patients selected for local therapy. In addition, preservation of hepatic parenchyma at the initial cytoreductive operation may have maintained liver reserve and expanded the feasibility of repeat liver-directed treatment at the time of progression.

The observed improvement in OS and the observed decrease in the proportion of patients undergoing major hepatectomy correlate with a transition in surgical strategy for NELM toward LPSC over time. Traditionally, surgical management of NELM focused on achieving maximal cytoreduction as a ≥ 90% reduction in tumor burden was long regarded as the benchmark for survival benefit.^6,7^ To meet this goal, major hepatectomy was often required.^8,9^ However, major hepatectomy may compromise hepatic reserve and preclude future interventions such as repeat resection or local ablative therapies, which is a problem given the high tumor progression rate in patients who undergo major hepatectomy and the long expected survival time in these patients.^10^ Subsequent studies identified residual tumor volume as a critical prognostic determinant, and accumulating evidence suggests that ≥ 70% cytoreduction can yield survival outcomes comparable to those observed with ≥ 90% resection.^11,12^ Historically, the effort to achieve maximal cytoreduction often led surgeons to perform major hepatectomy to clear small, deep, or bilobar lesions that would otherwise remain in the liver. However, if complete hepatic clearance is not necessary in every patient to produce oncologic benefit, as indicated by the newer studies, then extensive resection solely to remove all visible disease is not always required. Rather, parenchyma-sparing strategies, including limited resection with adjunctive ablation, may allow for meaningful cytoreduction while preserving future liver reserve and maintaining options for treatment at progression.^11,13^ The present study suggests that the reduced frequency of major hepatectomy may have contributed, at least in part, to the improvement in OS over time among patients with NELM, given that major hepatectomy remained associated with worse OS even after adjustment for treatment period. By preserving hepatic reserve, LPSC enables additional local and systemic treatments upon progression and may thus promote sustained long-term survival. The decreased use of concomitant ablation in the LP cohort may also reflect a change in the goal of cytoreduction over time. In the earlier period, ablation was often used as an adjunct to achieve complete or near-complete clearance of deep parenchymal lesions while preserving liver parenchyma. In the later period, as the acceptable threshold for cytoreduction became less stringent, ablation may have been used more selectively rather than routinely for all residual deep lesions.

During the study period, from 1998 through 2023, the paradigm of systemic therapy for NENs shifted from symptom control of functional tumors to suppression of tumor growth, a transition systematically supported by a series of landmark randomized controlled trials. SSAs became established as the standard first-line therapy for advanced NENs after the PROMID trial^14^ demonstrated a significant prolongation of time to tumor progression in midgut NENs (grade 1 and 2) treated with octreotide, and the CLARINET trial^15^ showed an unreached median PFS in nonfunctional gastrointestinal and lung NETs treated with long-acting SSAs. Around 2011, molecular targeted agents aimed at tumor growth signaling pathways were introduced for treatment of NENs. The mTOR inhibitor everolimus demonstrated PFS benefits in pancreatic NENs in the RADIANT-3 trial^16^ and in nonfunctional lung and gastrointestinal NENs in the RADIANT-4 trial.^17^ Sunitinib demonstrated efficacy in pancreatic NENs in a phase III randomized controlled trial,^20^ further expanding therapeutic options. PRRT, targeting somatostatin receptors, significantly improved PFS in patients with progressive midgut and pancreatic NENs in the NETTER-1 and NETTER-2 trials,^18,21^ establishing PRRT as another major pillar of systemic therapy.

In the present study, compared with the EP cohort, the LP cohort had significantly better OS and a higher proportion of patients receiving SSAs and molecular targeted therapy at the time of tumor progression. Although the study included patients with various primary tumor sites and the detailed effects of diverse systemic regimens introduced during the 25-year study period could not be fully adjusted for, most pivotal trials of systemic therapies for NETs have demonstrated improvements primarily in progression-free survival rather than definitive overall survival benefits. Therefore, the improved OS observed in the LP cohort may reflect not only the adoption of individual evidence-based systemic therapies after 2009, but also the cumulative effect of sequential systemic treatments combined with repeated local therapies.

There are several limitations to this study. First, this was a retrospective study conducted at a single institution, and potential biases in data collection could not be completely avoided. Second, systemic therapies for NENs evolved substantially over the 25-year study period, and given the heterogeneity of primary tumor sites among the patients in our study, it was not possible to fully adjust for the diverse treatment regimens introduced during this long study period. Third, percent cytoreduction was based on the surgeon’s assessment documented in the operative report, rather than systematic volumetric comparison of preoperative and postoperative imaging, which may have introduced measurement variability. Fourth, the high rate of achieving > 90% cytoreduction suggests that patients were selected for surgery when meaningful cytoreduction was considered feasible. Therefore, the present findings may not be generalizable to patients with higher tumor burden or more diffuse liver involvement who were not considered suitable candidates for surgical cytoreduction. Fifth, key clinicopathologic variables, including World Health Organization (WHO) grade and lymph node metastasis, were missing in a substantial proportion of patients, which may have limited adjustment for disease biology and introduced residual confounding. Finally, treatment selection at the time of disease progression (e.g., local therapy versus SSA) was likely influenced by selection biases such as tumor burden and patient’s general health status. Therefore, the association between local therapy and improved survival may, at least in part, reflect the better baseline status of patients who were eligible for local treatment.

## Conclusions

OS after hepatic cytoreduction for NELM improved during 1998–2023. This improvement was observed in parallel with the decreased use of major hepatectomy and increased adoption of parenchyma-sparing approaches, which preserve hepatic reserve and may allow for subsequent local therapies. The systematic introduction of evidence-based systemic treatments, including SSAs and targeted agents, also likely contributed. These findings support a shift from a goal of “≥ 90% cytoreduction” and major hepatectomy toward individualized surgical strategies that balance tumor reduction with preservation of liver function and future treatment options.

## Supplementary Information

Below is the link to the electronic supplementary material.

Supplementary file1 (DOCX 44 KB)

Supplementary file 2 (TIFF 16610 KB): Figure S1. Love plot showing covariate balance before and after propensity score matching. BMI, body mass index; G1, grade 1; G2, grade 2; G3, grade 3; NEC, neuroendocrine carcinoma; NET, neuroendocrine tumor; WHO, World Health Organization.

Supplementary file 3 (TIFF 16610 KB): Figure S2. Overall survival stratified by major hepatectomy and liver-parenchyma-sparing cytoreduction (LPSC) after propensity score matching.

## Abbreviations

CI: Confidence interval
EP: Earlier period
HR: Hazard ratio
IQR: Interquartile range
LP: Later period
LPSC: Liver-parenchyma-sparing cytoreduction
NELM: Neuroendocrine liver metastases
NEN: Neuroendocrine neoplasm
NET: Neuroendocrine tumor
OS: Overall survival
PFS: Progression-free survival
PRRT: Peptide receptor radionuclide therapy
SSA: Somatostatin analog
