# Supplementary material for: Changes in Outcomes of Hepatic Cytoreduction for Neuroendocrine Liver Metastases over Two Decades: From Major Hepatectomy to Parenchyma-Sparing Approaches
Source: Ann Surg Oncol. 2026 Aug 12;33(12):10013–21. doi: 10.1245/s10434-026-20383-z (PMC13675258; doi:10.1245/s10434-026-20383-z)
Supplement: Supplementary file 1 [file 10434_2026_20383_MOESM1_ESM.docx]

Supplementary Table 1. Multivariable Analysis of Progression-Free Survival^a^

| Variable | Univariable analysis | | | |  | | Multivariable analysis | | |
| --- | --- | --- | --- | --- | --- | --- | --- | --- | --- |
|  | HR | 95% CI | P value |  | | HR | | 95% CI | P value |
| Patient factors |  |  |  |  | |  | |  |  |
| Age, years | 1.00 | 0.99-1.02 | 0.408 |  | |  | |  |  |
| Sex, male:female | 1.04 | 0.80-1.35 | 0.78 |  | |  | |  |  |
| BMI, kg/m^2^ | 0.98 | 0.95-1.00 | 0.027 |  | | 0.99 | | 0.96-1.01 | 0.321 |
| Primary tumor factors |  |  |  |  | |  | |  |  |
| Primary lesion site |  |  |  |  | |  | |  |  |
| Pancreas | 1 (Ref) | - | - |  | | 1 (Ref) | | - | - |
| Small intestine | 0.61 | 0.47-0.79 | <0.001 |  | | 0.55 | | 0.37-0.81 | 0.003 |
| Other | 1.08 | 0.77-1.50 | 0.662 |  | | 0.77 | | 0.49-1.21 | 0.254 |
| Lymph node metastases | 1 | 0.69-1.46 | 0.999 |  | |  | |  |  |
| Liver tumor factors |  |  |  |  | |  | |  |  |
| Synchronous metastases | 0.88 | 0.66-1.16 | 0.365 |  | |  | |  |  |
| Extrahepatic metastases | 1.24 | 0.66-2.34 | 0.508 |  | |  | |  |  |
| Tumor number ≥2 | 1.37 | 1.02-1.85 | 0.035 |  | | 1.6 | | 1.08-2.37 | 0.019 |
| Maximum tumor diameter >5 cm | 1.31 | 0.97-1.76 | 0.074 |  | | 1.72 | | 1.16-2.55 | 0.007 |
| Pre-hepatectomy chemotherapy | 1.15 | 0.88-1.51 | 0.297 |  | |  | |  |  |
| Resection factors |  |  |  |  | |  | |  |  |
| Major hepatectomy | 0.85 | 0.65-1.10 | 0.213 |  | |  | |  |  |
| Concomitant ablation | 1.11 | 0.80-1.54 | 0.519 |  | |  | |  |  |
| Extent of resection |  |  |  |  | |  | |  |  |
| 100% | 1 (Ref) | - | - |  | | 1 (Ref) | | - | - |
| ≥90% | 1.61 | 0.99-2.61 | 0.054 |  | | 2.09 | | 1.19-3.66 | 0.01 |
| ≥70, <90% | 7.71 | 1.88-31.62 | 0.005 |  | | - | | - | - |
| <70% | 2.33 | 0.86-6.31 | 0.097 |  | | 2.81 | | 0.67-11.71 | 0.156 |
| WHO grade |  |  |  |  | |  | |  |  |
| Grade 1/2 NET | 1 (Ref) | - | - |  | | 1 (Ref) | | - | - |
| Grade 3 NET | 1.67 | 0.96-2.91 | 0.07 |  | | 1.21 | | 0.65-2.24 | 0.555 |
| NEC | 2.18 | 1.01-4.72 | 0.048 |  | | 2.11 | | 0.93-4.78 | 0.074 |
| Treatment period |  |  |  |  | |  | |  |  |
| EP (1998–2008) | 1 (Ref) | - | - |  | |  | |  |  |
| LP (2009–2023) | 1.04 | 0.80-1.35 | 0.766 |  | |  | |  |  |
| BMI, body mass index; EP, earlier period; LP, later period; NEC, neuroendocrine carcinoma; NET, neuroendocrine tumor; WHO, World Health Organization.  ^a^235 patients were included in the multivariate analysis of progression-free survival. | | | | | | | | | |

Supplementary Table 2. Multivariable Analysis of Overall Survival in Patients Treated for Tumor Progression^a^

| Variables | Univariable analysis | | | |  | | Multivariable analysis | | |
| --- | --- | --- | --- | --- | --- | --- | --- | --- | --- |
|  | HR | 95% CI | P value |  | | HR | | 95% CI | P value |
| Patient factors |  |  |  |  | |  | |  |  |
| Age, years | 1.03 | 1.00-1.05 | 0.017 |  | | 1.05 | | 1.01-1.08 | 0.012 |
| Sex, male:female | 0.91 | 0.59-1.39 | 0.651 |  | |  | |  |  |
| BMI, kg/m^2^ | 0.95 | 0.91-0.99 | 0.015 |  | | 0.94 | | 0.87-1.01 | 0.092 |
| Primary tumor factors |  |  |  |  | |  | |  |  |
| Primary lesion site |  |  |  |  | |  | |  |  |
| Pancreas | 1 (Ref) | - | - |  | | 1 (Ref) | | - | - |
| Small intestine | 0.6 | 0.38-0.95 | 0.03 |  | | 1.22 | | 0.45-3.30 | 0.691 |
| Other | 1.37 | 0.82-2.29 | 0.226 |  | | 1.38 | | 0.53-3.63 | 0.513 |
| Lymph node metastases | 1.16 | 0.60-2.23 | 0.652 |  | |  | |  |  |
| Liver tumor factors |  |  |  |  | |  | |  |  |
| Synchronous metastases | 1.16 | 0.74-1.83 | 0.516 |  | |  | |  |  |
| Extrahepatic metastases | 1.4 | 0.34-5.74 | 0.638 |  | |  | |  |  |
| Tumor number ≥2 | 1.25 | 0.76-2.06 | 0.386 |  | |  | |  |  |
| Maximum tumor diameter >5 cm | 1.61 | 1.02-2.55 | 0.041 |  | | 1.41 | | 0.63-3.14 | 0.401 |
| Pre-hepatectomy chemotherapy | 1.53 | 0.98-2.39 | 0.059 |  | | 1.92 | | 0.89-4.15 | 0.097 |
| Resection factors |  |  |  |  | |  | |  |  |
| Major hepatectomy | 1.41 | 0.92-2.17 | 0.11 |  | |  | |  |  |
| Concomitant ablation | 1.01 | 0.61-1.67 | 0.962 |  | |  | |  |  |
| 100% | 1 (Ref) | - | - |  | |  | |  |  |
| ≥90% | 0.54 | 0.17-1.70 | 0.292 |  | |  | |  |  |
| ≥70, <90% | 1.57 | 0.38-6.39 | 0.531 |  | |  | |  |  |
| <70% | 0.32 | 0.04-2.32 | 0.26 |  | |  | |  |  |
| WHO grade |  |  |  |  | |  | |  |  |
| Grade 1/2 NET | 1 (Ref) | - | - |  | | 1 (Ref) | | - | - |
| Grade 3 NET | 3.98 | 1.62-9.81 | 0.003 |  | | 5.46 | | 1.68-17.72 | 0.005 |
| NEC | 1.69 | 0.40-7.08 | 0.473 |  | | 1.39 | | 0.28-6.96 | 0.688 |
| Initial treatment for progression |  |  |  |  | |  | |  |  |
| Cytotoxic chemotherapy | 1 (Ref) | - | - |  | | 1 (Ref) | | - | - |
| Local therapy | 0.45 | 0.23-0.89 | 0.022 |  | | 0.41 | | 0.17-0.97 | 0.043 |
| Somatostatin analogue | 0.65 | 0.30-1.40 | 0.271 |  | | 0.72 | | 0.30-1.73 | 0.462 |
| Molecular targeted therapy | 0.48 | 0.26-0.90 | 0.023 |  | | 0.5 | | 0.21-1.21 | 0.126 |
| Other | 1.06 | 0.43-2.66 | 0.895 |  | | 0.64 | | 0.21-1.99 | 0.445 |
| Treatment period |  |  |  |  | |  | |  |  |
| EP (1998–2008) | 1 (Ref) | - | - |  | | 1 (Ref) | | - | - |
| LP (2009–2023) | 0.53 | 0.33-0.85 | 0.009 |  | | 0.68 | | 0.32-1.43 | 0.309 |
| BMI, body mass index; NEC, neuroendocrine carcinoma; NET, neuroendocrine tumor; WHO, World Health Organization.  ^a^112 patients were included in the multivariate analysis of overall survival. | | | | | | | | | |

Supplementary Table 3. Characteristics of patients by major hepatectomy after propensity score matching

| Characteristics | Before Matching | | |  | | After Matching | | | | |
| --- | --- | --- | --- | --- | --- | --- | --- | --- | --- | --- |
|  | Major hepatectomy  n = 129 | LPSC  n = 156 | P value | |  | | Major hepatectomy  n = 50 | LPSC  n = 50 | P value | |
| Patient factors |  |  |  | |  | |  |  |  | |
| Age, years | 55.1 (12.4) | 57.9 (11.3) | 0.043 | |  | | 55.8 (12.4) | 54.9 (11.7) | 0.702 | |
| Sex (male/female) | 64 (49.6) | 82 (52.6) | 0.706 | |  | | 23 (46.0) | 22 (44.0) | >0.999 | |
| BMI, kg/m2 | 27.7 (5.7) | 29.1 (6.6) | 0.06 | |  | | 28.5 (6.1) | 28.3 (6.1) | 0.87 | |
| Primary tumor factors |  |  |  | |  | |  |  |  | |
| Primary lesion |  |  | 0.035 | |  | |  |  | 0.829 | |
| Small intestine | 35 (27.1) | 23 (14.7) |  | |  | | 12 (24.0) | 11 (22.0) |  | |
| Pancreas | 39 (30.2) | 55 (35.3) |  | |  | | 19 (38.0) | 17 (34.0) |  | |
| Others | 55 (42.6) | 78 (50.0) |  | |  | | 19 (38.0) | 22 (44.0) |  | |
| Lymph node metastases | 70 (79.5) | 104 (82.5) | 0.708 | |  | | 25 (71.4) | 32 (80.0) | 0.551 | |
| Liver tumor factors |  |  |  | |  | |  |  |  | |
| Synchronous | 77 (67.0) | 92 (61.7) | 0.456 | |  | | 25 (55.6) | 33 (70.2) | 0.215 | |
| Extrahepatic metastases | 5 (3.9) | 7 (4.5) | >0.999 | |  | | 2 (4.0) | 1 (2.0) | >0.999 | |
| Tumor number | 5.0 (5.7) | 3.3 (3.8) | 0.003 | |  | | 4.8 (3.5) | 5.0 (5.5) | 0.845 | |
| Maximum tumor diameter, mm | 49.0 (35.0) | 29.7 (25.2) | <0.001 | |  | | 42.3 (31.4) | 39.7 (31.5) | 0.678 | |
| Pre-hepatectomy chemotherapy | 50 (38.8) | 48 (30.8) | 0.198 | |  | | 24 (48.0) | 18 (36.0) | 0.311 | |
| WHO grade |  |  | 0.123 | |  | |  |  | 0.937 | |
| G1/G2 NET | 34 (45.3) | 59 (50.0) |  | |  | | 25 (50.0) | 26 (52.0) |  | |
| G3 NET | 29 (38.7) | 51 (43.2) |  | |  | | 20 (40.0) | 20 (40.0) |  | |
| NEC | 12 (16.0) | 8 (6.8) |  | |  | | 5 (10.0) | 4 (8.0) |  | |
| Postoperative outcomes |  |  |  | |  | |  |  |  | |
| Overall morbidity | 61 (47.3) | 61 (39.1) | 0.267 | |  | | 22 (44.0) | 22 (44.0) | >0.999 | |
| Major morbidity, CD grade ≥3 | 25 (19.4) | 16 (10.3) | 0.064 | |  | | 5 (10.0) | 5 (10.0) | >0.999 | |
| Bile leak | 6 (3.8) | 3 (2.3) |  | |  | | 2 (4.0) | 1 (2.0) |  | |
| Postoperative bleeding | 6 (3.8) | 8 (6.2) |  | |  | | 0 | 2 (4.0) |  | |
| Liver failure | 0 | 1 (0.8) |  | |  | | 0 | 0 |  | |
| Others | 13 (8.3) | 4 (3.1) |  | |  | | 3 (6.0) | 2 (4.0) |  | |
| 90-day mortality | 2 (1.6) | 2 (1.3) | >0.999 | |  | | 1 (2.0) | 0 (0.0) | >0.999 | |
| Abbreviations: BMI, body mass index; CD, Clavien-Dindo; LPSC, liver-parenchyma-sparing cytoreduction; NEC, neuroendocrine carcinoma; NET, neuroendocrine tumor; WHO, World Health Organization. | | | | | | | | | |  |
